# Reevaluation of Historical Exposures to Ethylene Oxide Among U.S. Sterilization Workers in the National Institute of Occupational Safety and Health (NIOSH) Study Cohort

**DOI:** 10.3390/ijerph16101738

**Published:** 2019-05-16

**Authors:** Kenneth T. Bogen, Patrick J. Sheehan, Ciriaco Valdez-Flores, Abby A. Li

**Affiliations:** 1 Health Sciences, Exponent, Oakland, CA 94612, USA; kbogen@exponent.com (K.T.B.); abbyli@exponent.com (A.A.L.); 2 Department of Industrial and Systems Engineering, College of Engineering, Texas A&M University, College Station, TX 77843, USA; ciriacov@tamu.edu

**Keywords:** cancer risk, ethylene oxide, historical occupational exposure, industrial hygiene, sterilization facilities

## Abstract

The 2016 U.S. Environmental Protection Agency (EPA) Integrated Risk Information System (IRIS) assessment for ethylene oxide (EO) estimated a 10^−6^ increased inhalation cancer risk of 0.1 parts per trillion, based on National Institute of Occupational Safety and Health (NIOSH) epidemiology studies of sterilization facility workers exposed to EO between 1938 and 1986. The worker exposure estimates were based on a NIOSH statistical regression (NSR) model “validated” with EO levels measured after 1978. Between 1938 and 1978, when EO data was unavailable, the NSR model predicts exposures lowest in 1938 increasing to peak levels in 1978. That increasing EO concentration trend arose, in part, because engineering/industrial-hygiene (E/IH) factors associated with evolving EO-sterilization equipment and operations before 1978 were not properly considered in the NSR model. To test the NSR model trend prediction, a new E/IH-based model was developed using historical data on EO kill concentrations, EO residue levels in sterilized materials, post-wash EO concentrations in a sterilization chamber, and information on facility characteristics and sterilizer operator practices from operators familiar with pre-1978 industry conditions. The E/IH 90th percentile of 8 h time-weighted average EO exposures (C_90_) for highly exposed sterilizer operators was calibrated to match 1978 C_90_ values from the NSR model. E/IH model C_90_ exposures were estimated to decrease over time from levels 16 and were four-fold greater than NSR-estimated exposures for workers during 1938–1954 and 1955–1964. This E/IH modeled trend is opposite to that of NSR model predictions of exposures before 1978, suggesting that EPA’s exclusive reliance on the NIOSH cohort to estimate EO cancer risk should be re-examined.

## 1. Introduction

In 2016, the U.S. Environmental Protection Agency (EPA) Integrated Risk Information System (IRIS) completed a cancer risk assessment of ethylene oxide (EO), concluding that human lifetime chronic respiratory exposure to EO poses a unit risk of 9.1 per part per trillion (ppt) in relation to increased combined risk of lymphoid and breast cancer. The EPA assessment implied a cancer slope of 0.005 (µg/m^3^)^−1^, and that exposure to 0.1 ppt EO to the general population may increase this risk by one chance in a million [1]. EPA’s conclusion was based on its re-analysis of epidemiology data from an industry-wide cohort study of 18,254 sterilization workers, undertaken by the National Institute of Occupational Safety and Health (NIOSH). This cohort includes 1,222 sterilizer operators—a highly exposed job category—in 14 plants from 1938 to 1986, with an average of 4.9 years of EO exposure and 16.1 years of follow-up [2,3,4]. Those sterilizer operators experienced an average respiratory EO exposure of 4.3 ppm between 1976 and 1985 based on 627 8 h time-weighted average (TWA) personal air samples from 13 plants, with most collected in the 1980s, during or after the introduction of engineering controls to reduce worker exposure. NIOSH determined that these data were sufficient to estimate corresponding historical exposures. The NIOSH cohort averaged 26.8 years of follow-up in an extended follow-up study through 1998, by which time 16% of the cohort had died [5].

Log-transformed EO exposures for the NIOSH cohort were estimated using a NIOSH-developed statistical regression (NSR) model. The NSR model was “validated” using 2700 TWA charcoal tube measurements for workers’ personal breathing zones in 18 sterilization facilities (13 of which included workers represented in the NIOSH cohort). Virtually all measurements were obtained during 1978–1985, with only seven mean (derived from 23 total) measurements made during 1976–1977, and no measurements obtained before 1976 [6,7]. Most of the air samples were obtained in the 1980s, about the time that engineering controls and new work practices designed to lower worker exposure levels were introduced. The data were divided into two sets, one for developing the NSR model and the second for testing it. Of 23 independent variables tested for model inclusion, seven were determined to be significant (p ≤ 0.10) predictors of EO exposure and included in the final NSR model: job category, type of product sterilized, rear exhaust, aeration, days since product sterilization, sterilizer volume, and calendar year. Five of these variables were linear predictors, two (sterilizer volume and calendar year) were linear quadratic predictors, each representing inverted parabolic predicted contributions to exposure, with maximum predicted contributions at calendar year = 1978 and sterilizer volume = 1000 ft^3^. Sterilizer volume and calendar year were determined to have the greatest exposure predictivity for the period when measurements were also available [7].

**Figure 1:**
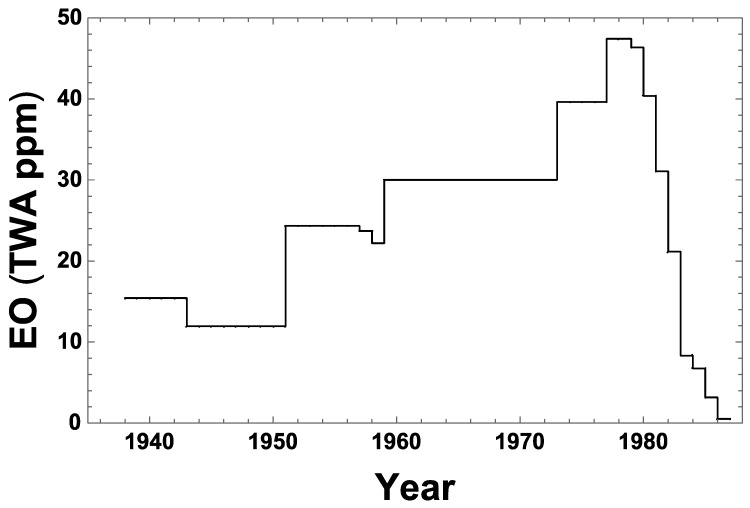
National Institute of Occupational Safety and Health (NIOSH) statistical regression (NSR) exposure model predictions of historical 90th percentile 8-h time-weighted average (TWA) occupational ethylene oxide (EO) concentration (C_90_) during 1936–1986, specific to all facilities and job categories addressed by that model. Nearly all of the facilities sterilized medical/health products. The NSR model predicts that C_90_ = 47.4, 30.0, and 11.9 ppm in 1978, 1959, and 1949, respectively, and predicts TWA C_90_ values of 34.3, 27.5, and 15.9 ppm during the late, middle, and early periods defined for the present study, respectively.

The resulting fitted NSR model explains 85% of the variation in measured TWA EO exposure levels used to validate the model [7]. However, this model was applied by NIOSH conditional on year = max (1978, calendar year), thus ignoring for the years before 1978 the inverse parabolic fit NIOSH obtained for available EO measurement data specifically in relation to calendar year. It was this conditional model that NIOSH applied to estimate exposure levels for all periods, facilities, and job categories [7]. Figure 1 plots the resulting NSR estimates of the annual 90th percentile values of occupational, 8 h TWA EO inhalation exposure (C_90_), relative to values of all facility and job category-specific EO exposure levels represented in the NIOSH cohort data set. This figure shows a clearly increasing EO concentration trend during 1938–1978, similar to the C_90_ trend estimated for NIOSH cohort cases calculated by EPA (USEPA 2016 [1], Appendix D-5, Figure D-23 on p. D 67). The increasing trend in estimated EO exposures during 1938–1978 was recognized by EPA as being due to two combined factors: sterilizer volumes tended to increase during that period, and measured (nearly all post-1977) EO levels were determined by NIOSH to fit best to an inverse parabolic function of sterilizer volume [1] (Appendix I, pp. I-26–I-27). However, these two factors are likely coincidental, insofar as they are not linked directly by data available during the earlier period. It was not the volume of each sterilizer used that determined total EO usage in a facility, but rather the capacity and utilization of all sterilizers combined in that facility. The EO concentrations experienced depended not only on the EO mass used, but also on the volumes and air turnover rates of rooms in which sterilization and storage of sterilized materials took place. It is reasonable to assume that during earlier decades when fewer sterilizers were operated using less total EO mass to treat smaller total masses of products, correspondingly smaller facilities were used for this purpose. Moreover, the NSR model did not directly address other substantial changes in pre-1978 sterilizer facilities and operations that may have reduced EO exposures during that period, when industrial hygiene EO data were either very limited (1976–1977) or unavailable (pre-1976), as a direct basis to verify pre-1978 NSR model predictions.

After reviewing a limited set of NSR-model predictions showing an increasing trend in predicted EO levels during 1938–1978, an EPA Science Advisory Board concluded that such “surprising historical behavior” was “unlikely” [8]. It was well recognized by others at NIOSH that before the late 1970s, EO exposures among sterilizer workers “were likely to have been higher … before installation of engineering controls, when the OSHA standard was 50 ppm” [2]. Industrial hygiene data collected in other industries indicate historical occupational exposure levels as well as industrial exposure limits for nearly all chemical toxicants show generally decreasing historical trends in exposure concentrations since the establishment of corresponding industrial exposure guidelines, such as the American Council of Government and Industrial Hygienists (ACGIH) threshold limit value (TLV) guidelines and regulatory limits such as OSHA regulations. For example, such trends are well documented for industries using perchloroethylene and/or trichloroethylene [9,10,11]. Such decreasing historical trends reflect improvements in technology sophistication, operational efficiency, knowledge concerning toxicological effects, and the implementation of workplace guidance values and standards. In the case of EO, such trends are reflected by the decrease in ACGIH 8 h TLVs for respiratory EO exposure over the period 1948–1984 [12] (see Table 1). Thus, the surprising historical pattern predicted by the NSR model indicates a need for closer examination.

A comprehensive re-examination of the intended basis of the NSR model is no longer possible, because the original data NIOSH used to develop that model no longer exist [1] (Appendix H, p. H-28). However, only a few personal air measurements were available during 1976–1977, and none were available before 1976. Consequently, this model’s surprising prediction that EO exposures generally increased historically through to 1978 remain effectively unvalidated. To better assess the reliability of pre-1978 NSR model predictions, an alternative engineering/industrial-hygiene (E/IH) factors-based model was developed, as described in the Materials and Methods section. This E/IH model uses historical data on measured EO residue levels in sterilized materials, post-wash EO concentrations in a sterilization chamber, and facility characteristics and sterilizer operator practices obtained by interviews with highly experienced former sterilizer operators, experts who were familiar with 1960s–1970s EO sterilization industry conditions. These interviews focused on EO-exposure conditions experienced by sterilizer operators, whose tasks during that period involved among the highest EO exposures relative to those of other sterilization industry workers in facilities represented in the NIOSH cohort. For this reason, the E/IH model focused on predicting C_90_ as a measure of EO exposure for such relatively highly exposed workers, and was calibrated to match the NSR model prediction of C_90_ in 1978, the first year in which the NSR model was empirically validated using a large number of average measures of EO in personal air. Key assumptions of the E/IH model were then modified, as explained in the Materials and Methods section, to reflect operating conditions expected to be pertinent to medical/health products sterilization during each of the two earlier periods of sterilization industry operations we considered (1938–1954 and 1955–1964). In addition, a spice products sterilization scenario during the latest period was also examined, to provide insight on the other primary product type that is sterilized. To assess the plausibility of the NSR model, E/IH model predictions were compared with corresponding NSR model predictions, as shown in Figure 1. The primary objectives of this modeling study were to examine whether any historical trend (increasing, decreasing, or constant) is exhibited in the EO exposure levels estimated by the E/IH approach before 1978 and to compare the consistency of any such trend with results produced using the NSR model.

## 2. Materials and Methods

### 2.1. E/IH Model Focus

Modeling for the NIOSH sterilization worker cohort focused on operations involving medical/health products sterilized during three periods of industry development before 1978: 1938–1954 (Early Period), 1955–1964 (Middle Period), and 1965–1978 (Late Period). These operations are listed in Box 1. The beginning years of the Middle and Late periods were set to reflect an approximate seven year lag, reflecting time to implement technological and operational improvements after adoption of the initial and second ACGIH 8 h TLV exposure levels for EO of 100 ppm in 1948 and 50 ppm in 1957 [12] (see Table 1). In addition, a typical Late Period spice-sterilization facility operation was modeled. There was inadequate data to model for spice product operations in earlier years, although exposure trends would likely have been similar to those for medical health products, as operating conditions are believed to have been similar. The selected modeling scenarios reflect that most of the 14 facilities in the NIOSH cohort primarily sterilized medical/health-related products and supplies with EO, whereas three of the plants “treated spices with ethylene oxide and another manufactured and tested sterilizers with ethylene oxide” [4]. The periods addressed include all of the pre-1978 years for which NIOSH had no (pre-1976) or relatively few (1976–1977) average measures of personal air EO.

Box 1 **Summary of EO Sterilization Operations (Activities Implemented to Conduct Historical EO Sterilization Operations).**

(1) Implanting efficacy test samples into selected boxes of materials on pallets to be sterilized
(2) Loading pallets of materials to be sterilized into a chamber
(3) Adding EO as a mixed or pure gas to the chamber to the desired level (kill concentration)
(4) Running the chamber under the desired temperature and relative humidity conditions for a duration to achieve the desired level of microbial mortality
(5) Exhausting some EO gas from chambers to in-room water traps (inefficient traps released EO into work room air)
(6) In some cases, implementing air/nitrogen washes to reduce remaining EO concentrations in chamber air and residue levels in sterilized materials before unloading the chamber (no or few washes were used during the time frame of interest)
(7) Unloading the processed materials from the chamber (released EO into work room air)
(8) Removing test samples from boxes for efficacy analysis (operators in close contact with off-gassing packaging and materials emitting EO into work room air)
(9) Transporting the sterilized materials to a storage area in or separate from the work room, and working in the storage area where materials remained until returned to customers (sterilized packaging and materials continue to off-gas during storage contributing additional EO to work room or storage room air)

### 2.2. E/IH Model Structure and Parameters

**Figure 2:**
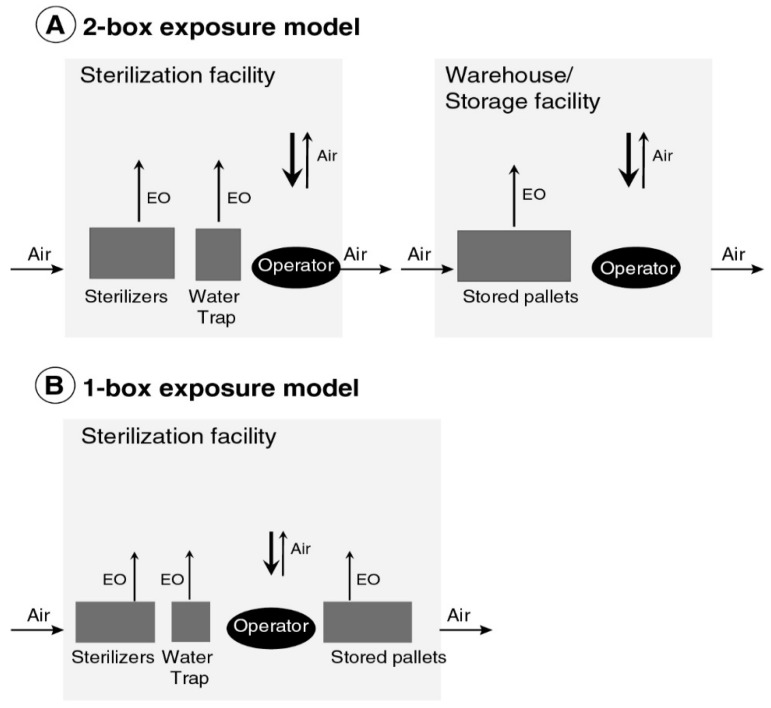
Engineering/industrial-hygiene (E/IH) model of occupational respiratory exposure to EO used to sterilize (**A**) medical/health products during the Late Period and (**B**) medical/health products during the Early and Middle periods, and spices during the Late Period.

During each of the three periods examined, C_90_ was estimated to represent sterilizer operator exposures using either of two compartmental E/IH model structures summarized in Figure 2, which were parameterized as summarized in Table 2 and Table 3. Based on information from interviews with three highly experienced former sterilizer operators who initially worked in the late 1960s and early 1970s, and a group of current EO sterilization experts who provided both information and written materials to support modeling, the E/IH model structures used imply that historical sterilization worker exposures resulted from:

1. accumulation of EO in a room in which sterilization operations occurred, resulting from emissions from chambers and water traps for exhausted gas;
2. accumulation of EO off-gassed from sterilized packaging as well as from treated materials, either stored in a separate warehouse or storage room (Figure 2A) or in the same room in which sterilization operations occurred (Figure 2B).

Specifically, to model EO exposure in the Late Period medical/health sterilization scenario, two unlinked compartments were used for modeling total exposure: one representing the building volume in which sterilization occurred and one representing a separate warehouse or storage building in which pallets of sterilized materials removed from sterilizers were stored before delivery. In this case, 8 h TWA exposure was calculated as a weighted average of separate TWA exposures estimated for each compartment, using as weights the respective average fractions of a sterilizer operator’s 8 h shift spent in each compartment (Figure 2A, Table 3). To model EO exposure in each of the three other scenarios considered (Early and Middle Period medical/health scenarios and Late Period spices scenario), a one compartment model was used to estimate exposures, assuming that all pallets of sterilized materials were stored in the same building in which sterilization operations also occurred (Figure 2B).

In sterilization areas, the percentage 100F_ch_% of all sterilizer operations associated EO exposure was assumed to arise from the mass (M_air_) of all post-cycle EO remaining in the chamber void space after the chamber doors were opened. The remaining percentage, 100(1–F_ch_)%, was assumed to arise from sterilizer leaks and periodic maintenance operations and from EO not captured by floor-level water-operated EO vapor traps. Many facilities used such traps to vent EO extracted from chambers during and/or just before opening a chamber door after completing the sterilization process [16]. The value of additional EO exposure was assumed to arise from the combined masses (M_prod_ and M_pack_) of EO released after being adsorbed/absorbed during sterilization into (1) sterilized products and (2) cardboard packaging (medical/health scenario) or bags (spices scenario) in which products were loaded onto pallets. Scenario-specific estimates of M_air_, M_prod_, and M_pack_ are listed in Table 3.

To estimate C_90_, the 8 h TWA concentration of EO for each E/IH-model compartment was evaluated at the dynamic equilibrium (at which, by definition, the rates of input and output are equal), using Equation (1):

(1) $$C_{90}=\frac{A}{1-F_{\mathrm{ch}}}H\frac{N\times M}{{\mathrm{AER}}_{\mathrm{eff}}\times V\times T}\frac{\mathrm{ppm}\;\mathrm{EO}}{1.8\;\mathrm{mg}/m^{3}},$$

in which N is the number of input units per shift; M is the input 90th percentile EO mass (mg) per unit of input (pallet or chamber) (M_air_ or (M_pack_ + M_prod_), depending on modeled input contribution); V is the compartment volume (m^3^); AER_eff_ is the expected effective unitless air exchange rate; T is the shift duration and averaging period (8 h); A = 1 if M is M_air_ or 0 otherwise (i.e., if M is M_pack_ + M_prod_); F_ch_ is the fraction of chamber-associated EO exposure assumed to arise from mass M_air_; and H is the EO concentration enhancement factor, due to the heavier-than-air density of EO.

Note that rates of dynamic (e.g., 1st order or 0 order) input do not appear in Equation (1), regardless of the complexity of the input patterns involved, provided that the air exchange rate (AER) is, on average, sufficiently large to ensure that dynamic equilibrium conditions are eventually closely approximated. The additional modeling assumptions and parameters listed in Table 2 and Table 3 were, as cited in these tables, based on relevant literature and/or interviews of sterilizer operators who began work in the late 1960s and early 1970s, as well as of other sterilization experts, who had detailed knowledge concerning earlier EO sterilizer industry facilities and operations. Information providing context for key modeling assumptions and parameters listed in Table 2 and Table 3 is discussed in the following subsections.

#### 2.2.1. Compartment Volumes

During the modeled Late Period operations for medical/health products, the interview-based estimates listed in Table 2 and Table 3 indicate a daily sterilization room volume/pallet ratio (VPR_SR_) equal to 11,000 m^3^ (19 pallets/day). The analogous ratio (VPR_WS_) for warehouse/storage areas was equal to 8500 m^3^ (19 × 3 × 7 pallets/day), assuming 19 pallets/shift, three shifts/day, and an average of seven days of treated pallet storage before delivery. The E/IH-model compartment volumes during the Early and Middle periods listed in Table 3 (for which no separate pallet storage area was assumed) were estimated as the product of (VPR_SR_ + VPR_WS_) and the assumed value of period-specific pallets/day, listed in Table 2. Thus, it was assumed that space utilization efficiency remained nearly constant over all modeled periods.

#### 2.2.2. Air Exchange Rate

**Figure 3:**
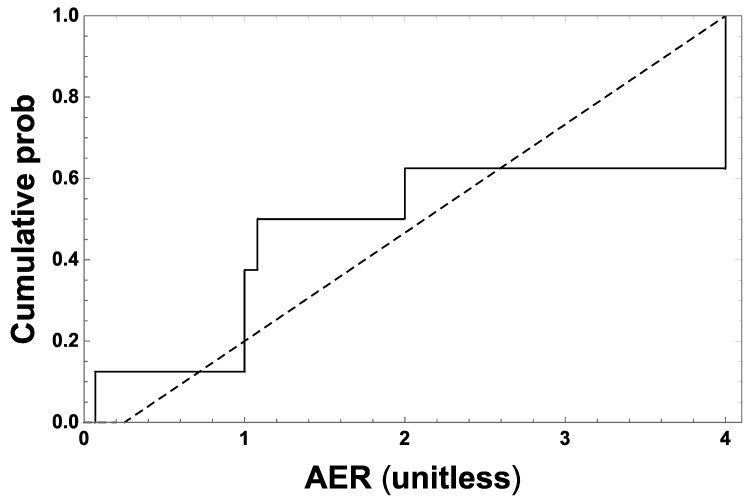
Empirical cumulative distribution of the subset of air exchange rate (AER) values reported for sterilization rooms in the spice sterilization facilities listed in Appendix C-10 of Goldgraben and Zank [16], for which AER ≤ 4/h, representative of sterilization rooms assumed not to have used forced-air ventilation. The data are consistent with an approximately uniform AER distribution between 0.25 and 4 per hour, as assessed (p = 0.52) by the Kolmogorov one sample test [26].

The air exchange rate (AER) applicable to sterilization and (if applicable) to separate warehouse/storage areas was assumed to be constant at the modeled facilities. Its estimated effective expected value (AER_eff_), applied over all modeled periods, was defined as the unbiased estimator in the multiplicative factor (1/AER_eff_) that appears in Equation (1). AER data for sterilization areas in 21 facilities that sterilized spices in the 1978–1981 period [16] indicate that forced-air ventilation was not widespread, even during that period and, where not used, was consistent with AER values approximately uniformly distributed between 0.25 and 4 per hour (Figure 3). This assumption implies that between these bounds, 1/AER has the cumulative probability distribution *F*(*x*) = (16 − 4/*x*)/15 and density *f*(*x*) = 4/(15 x^2^), and thus that AER_eff_ = 1/$\int_{1/4}^{4}x\;f\left(x\right)dx$ = 15/(4 ln(16))/h = ~1.35/h.

#### 2.2.3. EO Concentration in Sterilization Chambers

EO sterilization methods employed different concentrations of administered EO that varied over the periods considered. For example, the Mainz sterilization method involves a 10% EO:90% CO_2_ mixture at pressures of 3 to 6 atmospheres and temperatures of 35 to 65 °C, a 12% EO:88% halogenated hydrocarbon (e.g., freon) gas mixture introduced under pressure, and 100% EO introduced under vacuum [16,20,21,22,27]. Regardless of the EO formulation applied, substantial sustained chamber concentrations of EO ranging from 400 to 1200 mg/L—depending on duration, temperature, humidity, and product type—have always been required to achieve acceptable levels of sterilization. Sterilization efficacy was historically verified by recovering enclosed biological test samples placed into packaged materials on pallets before sterilization and removed after completion of the sterilization for quantifying microbial mortality [20,21,22,28]. It is assumed the period-specific final chamber concentrations of EO listed in Table 3 were derived to be consistent with the kill concentration range for EO just mentioned, by using EO concentrations measured in relation to the number of wash cycles applied (described below), and using a modern industrial sterilization chamber (Supplemental Materials, Figure S1).

#### 2.2.4. Wash Cycles

Since the 1980s, increasing numbers of repeated cycles of in-chamber, post-exposure vacuum and air- or nitrogen-washes, as well as post-treatment heating and aeration, have been applied in the sterilization process to drive down residual levels of EO in treated products and in workplace air. The wash cycle assumptions listed in Table 3 reflect the fact that two or fewer vacuum and air or nitrogen washes were used in earlier sterilization operations (e.g., Goldgraben and Zank [16]), resulting in higher residual EO levels in the chamber air and sterilized materials. For example, in the 1980s it was observed that a 98% reduction in post-sterilization residual EO could be achieved in treated medical products after 25 vacuum/wash cycles, however, at the end of EO exposure before such cycling, most (i.e., ~64%) EO was observed to be adsorbed/absorbed into packaging materials, whereas residual EO was observed predominantly (i.e., >80%) in/on treated materials after the application of multiple evacuation cycles, although specific residual values of retained EO mass were noted to be highly product dependent [23].

#### 2.2.5. Storage of Sterilized Materials

An important difference in the operating conditions of the periods is the storage of sterilized materials in the same room as the sterilization chambers in Early and Middle Period operations and storage in a separate warehouse room during Late Period operations (Table 3).

#### 2.2.6. Rate of EO De-Gassing

Approximate first-order rates of EO release from sterilized EO absorbent medical product materials are consistent with the corresponding approximate 2 h typical half-time of EO release (approximate range: 1–6 h) [21,22,24,25]. Similar rates of first-order EO loss from product sterilization of packaging materials (EO permeable plastic enclosures, cardboard boxes and separators, spice bags, etc.) were likely, whereas EO entries into sterilization room air from chamber leaks, chamber maintenance, and floor-trap areas were likely to have been either episodic and/or roughly continuous. However, EO release kinetics do not affect the E/IH-model prediction of long-term TWA EO concentration given by Equation (1).

#### 2.2.7. Fraction (F_ch_) of M_air_ Attributable to EO in Chamber Void

**Figure 4:**
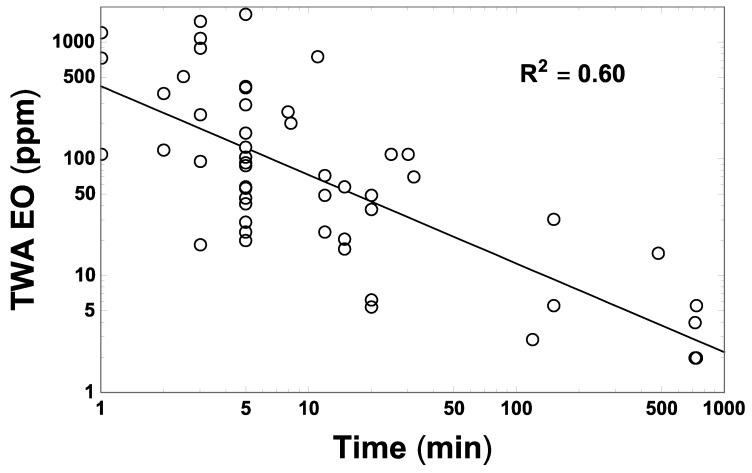
Fifty-five measures (open points) of TWA EO concentration made around sterilization chambers in the spice sterilization facilities listed and cited by Goldgraben and Zank [16], in relation to corresponding reported durations (*t*, in min) over which these measurements were made. The straight line shows the corresponding unweighted log–log linear regression fit to these data, EO_TWA_ = 419.65(*t*/min)^−0.7592^ ppm (*p* = ~0, R^2^ = 0.60), which predicts an 8 h TWA EO concentration of approximately 3.9 ppm.

The values of F_ch_ used in Equation (1) (namely, 0.44 and 0.39 for medical/health and spice product scenarios, respectively) were selected to predict a contribution to the overall 8 h TWA EO concentration attributable specifically to M_air_. This is consistent with that (~3.9 ppm) predicted by the log–log linear regression fit obtained to 55 TWA EO concentration measures discussed by Goldgraben and Zank [16], which were made in spice sterilization facilities over various averaging periods (Figure 4).

#### 2.2.8. EO Concentration in Worker Breathing Zone Due to EO Density

EO has a relative gas density of 1.5 compared to air. The experienced EO operators who were interviewed reported clear recollections of relatively greater EO concentrations at lower vertical positions, including recollections that had been confirmed by the EO concentration measurements taken, e.g., in low versus high positions in a storage warehouse. Using X and Y to denote EO mass in the lower and upper halves of each relevant compartment volume, and assuming sterilization operators were exposed to EO concentrations representative of those in the lower half of each compartment, it was conservatively assumed that the rate of vertical-diffusion gain of EO mass to X is 1.5-fold that of loss from Y. At dynamic equilibrium, the average EO concentration C_TWA_ = (X + Y)/2 was thus assumed to equal (X + X/1.5)/2 or (5/6)X, implying that X = (6/5)C_TWA_. Thus, the factor H in Equation (1) was assumed to be 6/5.

### 2.3. E/IH Model Calibration

Predictions made by the E/IH model for the Late Period medical/health products scenario were calibrated as follows to facilitate the comparison of predictions made using the E/IH and NSR models. Specifically, the 7% EO retention listed in Table 3, assumed to be applied after two vacuum/wash cycles, was selected to predict a value of C_90_ for the Late Period medical/health scenario that matches (to within ±0.5 ppm) the 47.4 ppm estimated by the NSR model [5,29,30]. This calibration enabled a direct, normalized comparison between predictions made by the E/IH and NSR models for the Early and Middle periods. E/IH estimates so generated were used to examine the plausibility of the pre-1976–1978 NSR predictions that could not be validated empirically by NIOSH because no personal air measurements were available. For the Early and Middle periods, EO retention was additionally assumed to vary in inverse proportion to the number of cycles assumed to be applied during those periods (Table 3).

### 2.4. Model Prediction Uncertainty

The interview- and literature-derived data used to construct period-specific E/IH-model assumptions for the input variables described above and their associated correlations were limited in scope and quantitative detail. For this reason, a Monte Carlo approach to quantifying the uncertainty associated with the C_90_ estimates generated by the E/IH model based on quantitative characterizations of uncertainty and variability associated with each model input was not feasible. Instead, C_90_ uncertainty for the year 1978 was estimated directly from the empirical distribution (*F*_C_) of 1806 facility and job category-specific estimates of occupational EO exposure estimated by the NSR model to have occurred during that year [29,30], which was the first year for which NSR model estimates could be validated, based on a large set of contemporary measurements of EO concentrations in personal air (Supplemental Materials, Figure S2). Ninety-five percent confidence bounds on the NSR-model value of C_90_ for the year 1978 were estimated robustly as corresponding maximally symmetrical 95% lower and upper confidence bounds (C_90Lo_ and C_90Hi_, respectively) on C_90,_ estimated from 10,000 bootstrap sets of 100 random samples generated from *F*_C_ as a parent distribution. The uncertainty in the value of C_90_ generated by the E/IH model for medical/health products sterilized during the Late Period (including the year 1978) was thus assumed to characterize the relative magnitude of C_90_ uncertainty for this scenario via corresponding lower- and upper-bound ratios *R*_Lo_ = C_90Lo_/C_90_ and *R*_Hi_ = C_90Hi_/C_90_, respectively. The same ratios were also used to estimate the relative magnitude of C_90_ uncertainty estimated by the E/IH model for each period and the sterilization product type considered. The decision to project C_90_ uncertainty specific to 1978 onto earlier periods, instead of using a similar approach applying NSR-based *F*_C_ distributions calculated using NIOSH summary data obtained for earlier years or sets of years, was made for three reasons. First, NSR-based estimates of occupational EO exposure before 1978 are potentially seriously biased. Very limited (1976–1977) or no (pre-1976) measured EO concentration data were available to validate pre-1978 historical estimates of worker exposures to EO generated by the NSR model. This lack of a substantive, contemporaneous empirical basis for the NSR model is also indicated by its failure to consider the historical evolution of specific engineering and industrial hygiene aspects of sterilization operations. Second, NSR-based estimates of upper-bound occupational EO exposure before 1978 are subject to increasingly serious sample-size bias for increasingly earlier years before 1978. The number of unique concentration estimates generated by the NSR model to separate facilities and job categories included in its set of NIOSH-cohort occupational exposure information was substantially less in earlier year (Supplementary Materials, Figure S3). Third, there is substantial uncertainty associated with extrapolating EO exposures for years and work conditions before data collection and sterilization industry regulation, using only a model fit to data and work conditions following industry regulations.

All calculations were performed using *Mathematica*^®^ 11.3 software (Wolfram Research, Inc., Champaign, IL, USA) [31].

## 3. Results

**Figure 5:**
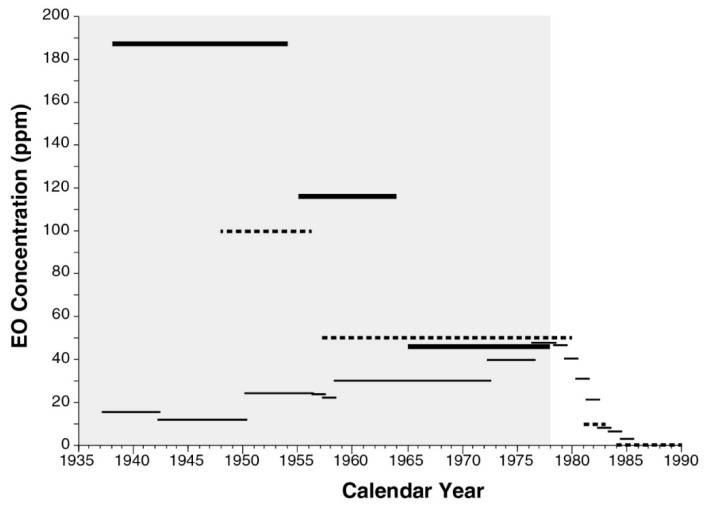
Comparison of E/IH (thick lines) and NSR (thin lines) exposure model estimates of occupational respiratory exposures to EO in facilities that sterilized medical/health products and prevailing ACGIH TLV limits for EO (dashed lines). Shaded area represents the period during which very limited or (pre-1976) no contemporaneous measurements were available to validate NSR model predictions and during which no EO-specific regulations were in place to limit occupational EO exposures.

The C_90_ estimates obtained using the E/IH modeling approach are summarized and compared in Table 4. The E/IH model results for medical/health product sterilization show an overall decreasing historical trend in estimated C_90_ during the period 1938–1978, with exposures in the Early Period higher than in 1978. The approximate values of C_90_ estimated by the E/IH model decrease from 190 to 120 to 48 ppm going forward in time from the Early to Middle to Late medical/health exposure periods considered. The former two E/IH-based estimates of C_90_ are ~3.9- and ~2.5-fold greater than that estimated for 1978 by both the E/IH and NSR models. In contrast, the NSR model predicts approximate C_90_ values of 12 and 30 ppm at the Early and Middle Period midpoints, respectively, implying that the C_90_ values predicted for the Early and Middle periods by the E/IH model for medical/health product sterilization are approximately 16- and 4-fold greater than those estimated by the NSR model, respectively (Table 4). The decreasing historical trend in occupational EO exposure levels predicted by the E/IH model during 1938–1978 contrasts sharply with the increasing trend predicted by the NSR model over this period. These contrasting trends are compared together with prevailing ACGIH TLV levels in Figure 5. The E/IH model generated very similar estimates of C_90_ for spice sterilization (not calibrated) and for medical/health product sterilization during the Late Period.

Using the bootstrap approach described in the Materials and Methods section, ratios *R*_Lo_ = 0.663 and *R*_Hi_ = 1.20 were calculated to estimate the relative magnitude of uncertainty in C_90_ (Table 4).

## 4. Discussion

The E/IH model estimated greater EO exposure concentrations in the Early and Middle periods examined relative to the concentrations it estimated for the Late Period. This decreasing historical trend over the period 1938–1978 is associated with a corresponding evolution in sterilization facility and operational characteristics, as reflected in the E/IH model parameter values and operational assumptions listed in Table 3. EO releases from the storage of sterilized products is predicted by the E/IH model to have been the primary contribution to total EO concentrations in workplace air over the periods examined. The two primary variables contributing to high EO residues in stored products during the Early and Middle periods are estimated to have been (1) the application of no or one vacuum and air/nitrogen wash and (2) the fact that sterilized product storage and sterilization operations typically occurred in the same building during those periods.

The NSR and E/IH models clearly predict opposite historical C_90_ trends, with the E/IH model predicting C_90_ values more than an order of magnitude greater than the NSR model during the Early Period considered for the sterilization of medical/health products. The E/IH model was calibrated to predict the same level of EO exposure (~48 ppm) during 1978, the end of the modeled Late exposure period. However, at midpoints of the Early and Middle exposure periods examined, the E/IH model estimated EO concentrations to be approximately 16- and 4-fold greater, respectively, than those predicted by the NSR model that was applied by NIOSH and EPA to estimate EO cancer risks based on analyses of NIOSH cohort epidemiological data. The substantial difference in the estimated historical trend of workplace EO concentrations during 1938–1978 between these two models clearly reflects the NIOSH model assumption that there were no substantive changes in sterilization operations before 1978, as well as an apparently spurious correlation imposed by that model between increased sterilizer volumes and its predicted EO concentrations during 1938–1978.

Results using the E/IH modeling approach reported here highlight the importance of considering combined sterilization and sterilization product storage in Early and Middle Period operations versus separate storage of sterilized materials in Late Period operations, which was not considered explicitly in the NSR model. These results clearly challenge the validity of NSR-based estimates of historical EO exposures, and thus challenge previous claims that the NSR model had been validated for this historical period [1,2,3,4]. Consequently, the results reported here imply that previous calculations of the elevated cancer risk experienced by members of the NIOSH sterilizer worker cohort [1,2,3,4] have greater uncertainty than had been described in those studies, insofar as those calculations presumed the validity of NSR model estimates of pre-1978 historical EO exposures experienced by members of that cohort.

## 5. Conclusions

In view of the uncertainty that the E/IH modeling analysis raises about NSR model predictions for early sterilization worker EO exposures, EPA’s exclusive reliance on the NIOSH cohort to estimate EO cancer potency and risk should be re-examined.

## Figures and Tables

**Table 1:** Historical summary guidance values and standards for EO exposure.

Date Range	Source *^a^*	Acceptable EO Concentration (ppm)
1948–1956	ACGIH TLV-TWA	100
1957–1980	ACGIH TLV-TWA	50
1978 (proposed)	FDA Maximum Residue Level	5–250
1981–1983	ACGIH TLV-TWA	10
1984–present	ACGIH TLV-TWA, A2 suspected human carcinogen	1
1984–present	OSHA PEL-TWA	1
2012–present	EC SCOEL implied 10^−6^ risk	0.0015
2016–present	EPA IRIS estimated 10^−6^ risk	0.0000001

*^a^* ACGIH TLV-TWA = American Conference of Governmental and Industrial Hygienists Threshold Limit Value-Time Weighted Average (8-h) [12]; FDA = U.S. Food and Drug Administration [13]; OSHA PEL-TWA = U.S. Occupational Safety and Health Administration Permissible Exposure Limit-Time Weighted Average [14]; EC SCOEL = European Commission Scientific Committee on Occupational Exposure Limits [15]; EPA IRIS = U.S. Environmental Protection Agency Integrated Risk Information System [1].

**Table 2:** Scenario assumptions applied for all modeled periods.

Model Parameter *^a^*	Sterilized Product Type	
	Medical/Health	Spices
Chamber, pallet volume per pallet	3.0, 2.0 m^3^/pallet *^a^*	18, 12 m^3^/chamber *^a^*
Void fraction	1/3 *^a^*	1/3 *^a^*
Air exchange rate (AER) *^b^*	0.25–4.0/h	0.25–4.0/h
Container/packaging mass	1.5 kg/box *^a^*^,*c*^	0.5 kg/bag *^a^*
Total container/packaging mass	24 kg/pallet *^d^*	48 kg/chamber
EO ad/absorbed in packaging, and in TM	10%, 2% *w*/*w* *^e^*	10%, 2% *w*/*w* *^e^*
TM mass	300 kg/pallet *^f^*	4,800 kg/chamber *^f^*

*^a^* Interviews with operator/managers with ≥30 years of detailed knowledge concerning sterilizer-industry facilities and operations. TM = sterilization-treated materials mass. *^b^* For sterilization operations room and if/as applicable also for warehouse (i.e., storage area); estimated from data in Appendix C-10 of Goldgraben and Zank [16] and assumed to be approximately uniformly distributed (see Materials and Methods and Figure 3). *^c^* [17]; *^d^* Estimated total cardboard/paperboard mass per pallet including internal paperboard spacing and/or packaging materials. *^e^* [18]. Container/packaging EO absorption assumed to be five-fold greater than that of TM. *^f^* Assumed effective TM density accounting for packing void space(s) = 0.3 kg/L (medical health), 0.4 kg/L (spices).

**Table 3:** Scenario assumptions for specific periods of E/IH model application.

E/IH Model Assumption *^a^*	Early Period (1938–1954)	Middle Period (1955–1964)	Late Period (1965–1978)	
	Medical/Health	Medical/Health	Medical/Health	Spices
Commercial operation	Starting (small rooms for chambers and material storage)	Expanding (larger rooms for chambers and material storage)	Further expanding (large rooms for chambers)	Further expanding (large rooms for chambers)
Sterilizer operation	Manual	Manual	Manual and automated	Manual and automated
Chamber size	Small (self-manufactured)	Small or mixed	Mixed size or large	Mixed size or large
Chamber operation	Under pressure	Under pressure	Under pressure or vacuum	Under pressure or vacuum
Loading/unloading	Hand	Pallet jack	Pallet jack	Pallet jack
Separate rooms for sterilization versus storage	No	No	Yes (no aeration)	No
Vacuum/wash cycles per treatment	0 *^a^*	1 *^a^*	1–2 *^a^*	2 *^b^*
Final EO concentration in sterilizer chamber air *^c^*	17,800 ppm	17,200 ppm	11,500 ppm *^c^*	5560 ppm
Sterilizer size and number: *^a^* #sterilizers/#palettes	4/2	9/2	2/2 + 3/3 + 1/6	2/6
Sterilization (warehouse/storage) room volume	4800 (NA) m^3^ *^d^*	11,600 (NA) m^3^ *^d^*	11,000 (8500) m^3^ ^*a*^	3100 (NA) m^3^ *^b^*
Hours per shift	8	8	8	8
Min/day in sterilization (warehouse/store) room	480 (NA)	480 (NA)	280 (180) *^a^*	480 (NA) *^b^*
Residual EO in sterilizer chamber air (M_air_)	32.0 g	31.0 g	20.4 g	59.4 g
Total packaging mass	24 kg/pallet	24 kg/pallet	24 kg/pallet *^c^*	48 kg/chamber
% EO retained post-cycle by packaging and TM *^e^*	21% *w*/*w*	14% *w*/*w*	10.5% *w*/*w*	7% *w*/*w*
EO retained by packaging (M_pack_)	504 g/pallet	336 g/pallet	252 g/pallet	67.2 g/chamber
EO retained by TM (M_prod_)	1260 g/pallet	840 g/pallet	630 g/pallet	14.4 kg/chamber

*^a^* Interviews with operator/managers with ≥30 years of detailed knowledge concerning sterilizer-industry facilities and operations and Perkins [19,20]; Bruch [21,22]; Goldgraben and Zank [16]; Buonicore et al. [23]. TM = sterilization-treated materials mass, NA = not applicable (stored where sterilized). No personal protective equipment (PPE) used during any period; limited worker protection controls only during the Late Period. *^b^* Goldgraben and Zank [16] (Appendix C-10), see Supplemental Materials, Table S1. *^c^* Measured values using a commercial sterilizer in 2018, assuming the cycle number value listed in row 1 (see Supplemental Materials, Figure S1). For EO, 1 ppm = 1.8 mg/m^3^. *^d^* Extrapolated from Late/Medical/health scenario assumptions (see Methods). *^e^* Estimated from Bruch [21,22]; Stetson et al. [24]; and White [25].

**Table 4:** Comparison of E/IH and NSR model results.

Scenario	Products Sterilized	Period	Model				
			NSR		E/IH		
			90th Percentile 8-h TWA EO Concentration, C_90_ *^a^* (ppm)	C_90_/(C_90_ Scenario 3) (ppm)	90th Percentile 8-h TWA EO Concentration, C_90_ *^a^* (ppm)	C_90_/(C_90_ Scenario 3) (ppm)	Fraction Due to Storage
1	Medical/health	Early	11.9 (7.90, 14.3)	0.33	187 (124, 224)	3.9	0.97
2	Medical/health	Middle	30.0 (19.9, 36.0)	0.58	118 (78.2, 141)	2.5	0.96
3	Medical/health	Late	47.4 (31.4, 56.8)	1.0	47.8 (31.7, 57.3)	1.0	0.95
4	Spices	Late	–	–	46.7 (31.0, 56.0)	0.98	0.92

*^a^* 95% confidence bounds listed in parentheses (see Methods). – = not applicable. For the NSR model, the Early and Middle periods were evaluated at period-specific midpoints (in 1946.5 and 1960, respectively), and the Late Period was evaluated in 1978 (see Figure 1). The E/IH to NSR ratios of C_90_ estimates listed for the Late, Middle, and Early periods are approximately 1.0, 3.9, and 16, respectively.

## Supplementary Materials

The following are available online at https://www.mdpi.com/1660-4601/16/10/1738/s1: Figures S1–S4, Table S1, and supporting references.
