# Supplementary material for: Reevaluation of Historical Exposures to Ethylene Oxide Among U.S. Sterilization Workers in the National Institute of Occupational Safety and Health (NIOSH) Study Cohort
Source: Int J Environ Res Public Health. 2019 May 16;16(10):1738. doi: 10.3390/ijerph16101738 (PMC6572526; doi:10.3390/ijerph16101738)
Supplement: Supplementary file 1 [file ijerph-16-01738-s001.pdf]

International Journal of
*Environmental Research and Public Health*

MDPI

# EO Supplementary Materials

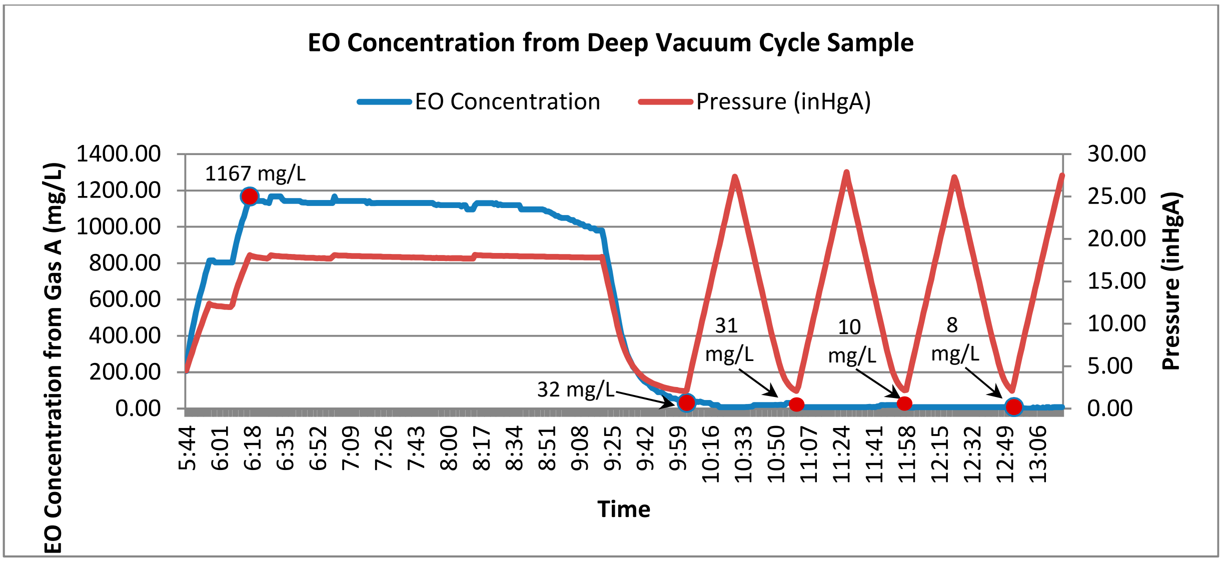


**Figure S1.** Ethylene oxide (EO) concentration in the chamber volume of a proprietary commercial sterilizer injected with 100% EO in relation to time, after application of 0–4 deep-vacuum cycles and subsequent corresponding air washes. The indicated residual EO concentrations are equivalent to 17,800, 17,200, 5560, and 4,440 ppm, respectively.

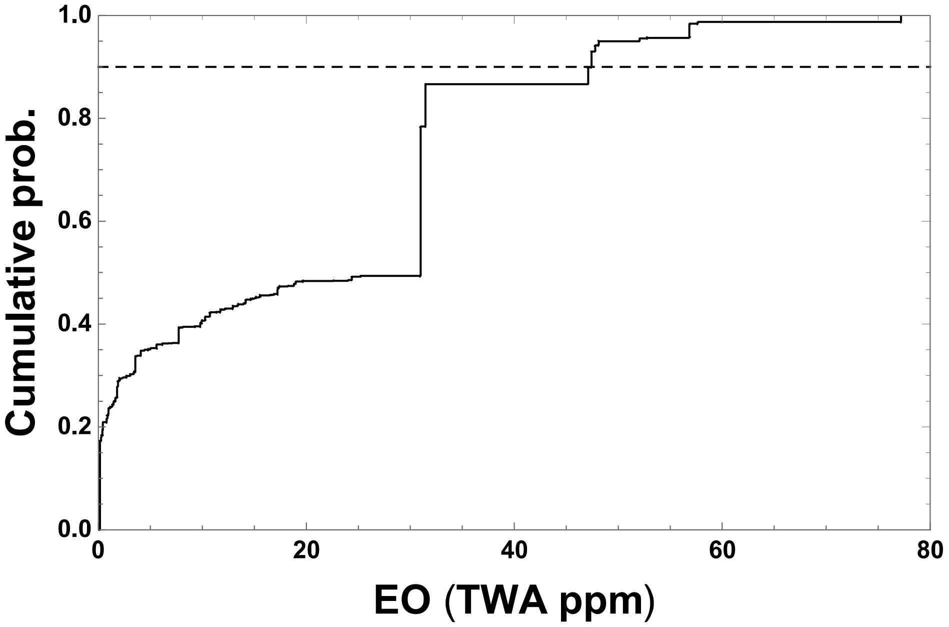


**Figure S2.** Cumulative distribution of NSR-model estimates of facility- and job-category-specific 8-hour TWA EO exposures in the NIOSH cohort of sterilizer workers during the year 1978 ($n$ = 1,806), the first year for which such estimates were validated based on a large sample of personal-air EO measurements [29,30]. Dashed line shows 90th percentile.

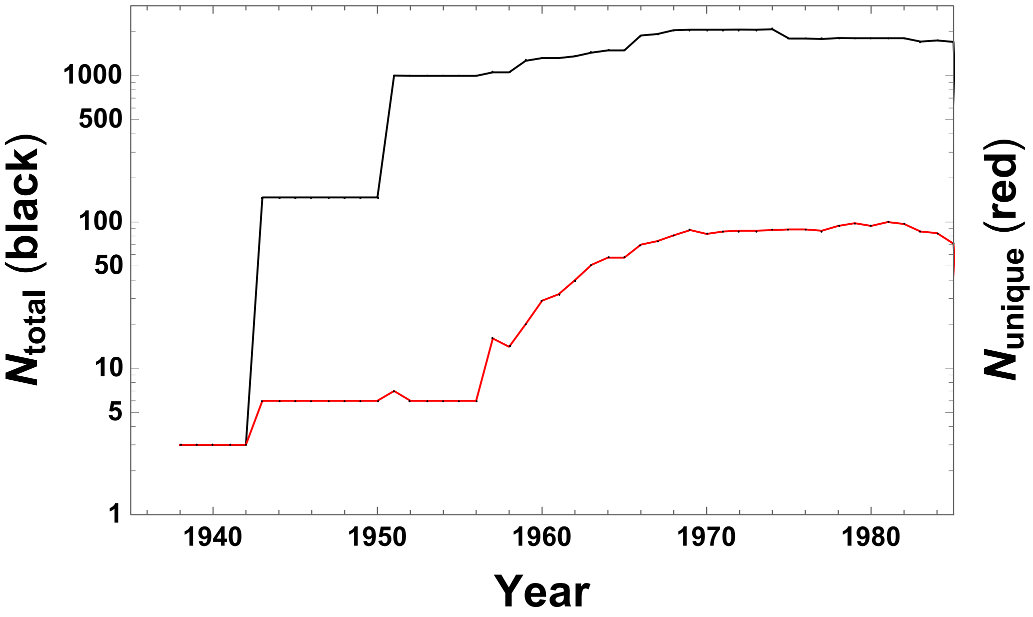


**Figure S3.** Total and unique numbers ($N_{total}$ and $N_{unique}$, respectively) of NSR-model facility- and job-category-specific estimates of EO concentration (reported with three decimal digits of precision) to which NIOSH cohort members were exposed from 1938 to 1985 (data source: NIOSH [30]; Lehman [29]).

**Table S1**. Practices Relevant to Worker Exposure at 21 Spice Industry Sites in ~1978-1981.

| Row | Site | Vstz (m3) | Nstz | Agent | Vent | Topen (h) | Stor | Tstor | Nvac | Vste (m3) | AERste | Vsto | AERsto |
|---|---|---|---|---|---|---|---|---|---|---|---|---|---|
| 1 | A | 10.87 | 1 | 100 | S | 0 | Y | NS | 2 | 152.9 | 15.56 | NS | NS |
| 2 | B | 7.22 | 2 | 12 | SA | 1 | N | 0.25 | 1 | 815.5 | 4 | NS | NS |
| 3 | C | 8.83 | 1 | 12 | A | 2 | Y | 1.5 | 2 | 2038.8 | 1 | NS | R |
| 4 | D | 28.25 | 2 | 100 | S | 0.75 | Y | 5 | NS | 732.7 | 10.05 | NS | NS |
| 5 | E | 15.09 | 1 | 12 | SA | 0 | N | 5 | 3 | 4893.2 | 1.08 | NS | NS |
| 6 | F | 15.09 | 1 | 12 | A | 0.75 | N | NS | 2 | 2038.8 | 23.33 | NS | NS |
| 7 | G | 15.86 | 1 | 100 | S | 0 | Y | NS | 2 | 453.1 | 5.62 | NS | NS |
| 8 | H | 9.34 | 1 | 12 | S | NS | Y | NS | 1 | 199.4 | 10 | NS | R |
| 9 | I | 34.69 | 4 | 100 | S | 0.17 | Y | NS | 3 | 603.1 | 14.09 | NS | 4 |
| 10 | J | 7.93 | 1 | 12 | A | 0.33 | Y | NS | 2 | 2293.7 | 0.07 | NS | 4 |
| 11 | K | 7.45 | 1 | 12 | S | 0.33 | NS | NS | 3 | 1962.4 | 4 | NS | NS |
| 12 | L | 26.08 | 1 | 12 | SA | 0.5 | N | 2 | 3 | 11326.7 | 2 | NS | NS |
| 13 | M | 18.41 | 1 | 12 | SA | 0.51 | N | 2 | 3 | 11326.7 | 1 | NS | R |
| 14 | N | 7.96 | 1 | 12 | A | 0.33 | NS | NS | 2 | 1019.4 | 5 | NS | R |
| 15 | O | 11.19 | 1 | 100 | S | 0.17 | N | NS | 2 | 224.3 | 30 | NS | NS |
| 16 | P | 7.05 | 1 | 100 | NS | NS | Y | NS | 3 | 1186.9 | 12.17 | NS | NS |
| 17 | Q | 3.4 | 1 | 12 | A | NS | N | NS | 3 | 672.8 | 2 | NS | NS |
| 18 | R | 39.64 | 1 | 100 | S | 0.17 | N | NS | 3 | 815.5 | 10.42 R | NS | 4 |
| 19 | S | 45.31 | 1 | 100 | SA | 0.17 | N | NS | 3 | 679.6 | 12.5 R | NS | 4 |
| 20 | T | 31.15 | 1 | 100 | SA | 0.17 | N | NS | 3 | 611.6 | 13.89 R | NS | 4 |
| 21 | U | 31.15 | 2 | 100 | SA | 0.5 | N | Y | 1 | 380.6 | 4 | NS | NS |
| MEDIAN | | 15.09 | 1 | ~50% ea. | ’66% to sewe | 0.33 | N | | 2.5 | 815.5 | | | |
| | | | | | | | | | | 2115.6 | Average | | |
| | | | | | | | | | | 3071.39 | Average Stor = N | | |

**Notes**

Source: Goldgraben R, Zank N. 1981. Mitigation of Worker Exposure to Ethylene Oxide. Report prepared for the U.S. Environmental Protection Agency. MTR-80 W333, March 1981. The Mitre Corp., McLean, VA. Appendix C-10: Practices Relevant to Worker Exposure at Spice Industry Sites, pp. C153-C175.

stz = sterilizer

ste = sterilization room

sto = storage room

Agent = P (= P% EO)

Vent = S (sewer drain), A (atmosphere), SA = both

Topen = hr after end of sterilization cycle at which chamber door opened
(0.51 if noted that operator not present when door opens)

Stor= Separate storage room for treated pallets (Y=Yes, N=No)

Tstor = treated pallet storage duration (d); "short" estimated as 0.25 d

Nvac = # vacuum and/or air-wash cycles (max)

Vx = vol (x = stz, ste, or sto) reported in ft3 if unspecified (or in specified unit)---- here converted to m3 (see below)

AER = air exchange rate reported in cu ft/min if (cfm) if unspecified; per unit time if time unit specified; ---- here converted to fraction/hr (see below)
Open or Cross-Ventilated= 4/h, Passive= 2/h, R = PPE including respirator used

NS = not specifed (or not applicable if Stor = N)

Median numeric height (18 ft) over all sites is listed for Site B for which height was not reported

Reported ft3 here converted to m3; reported AER here converted to 1/hr

## Average Value of a Linear-Model Single Compartment at Dynamic Equilibrium

A 1st-order (linear) input-output model $X(t)$ for which $\mathrm{d}X(t)/\mathrm{d}t = A\, k_{in}\, e^{-k_{\mathrm{in}} t} - k_{\mathrm{out}}\, X(t)$ has the solution $X(t) = k_{in}\, A(e^{-k_{\mathrm{out}} t} - e^{-k_{\mathrm{in}} t})/(k_{in} - k_{out})$, or $A\, k\, e^{-k\, t}$ if $k_{in} = k_{out} = k$, implying a corresponding $X(t)$ integral from $t = 0$ to $\infty$ (i.e., "area under the curve" or AUC) equal to $A/k_{\mathrm{out}}$ independent of $k_{in}$. In the case of repeated instantaneous inputs $A$ at intervals $t_A$, with each input subject to loss at 1st-order rate $k$, at time $t$ after the $n^{\mathrm{th}}$ input $X_n(t) = (A - X_{n-1}(t))e^{-k\, t} =$ $A(1-e^{-k\, t})/(e^{k\, t}-1)$, from which it can readily be shown that in the limit as $n \to \infty$ (i.e., at dynamic equilibrium) the TWA value of $X_n(t)$ over time $t = 0$ to $t = T$ is the input-to-output ratio $A/(k\, T)$, as required for any linear time-invariant closed (e.g., mass-conserving) system, including any one-compartment model with multiple inputs and outputs that occur at 1st-order rates $k_{in}$ and $k_{out}$, respectively, as long cited and proven mathematically in the case of periodic inputs at equal time intervals (e.g., Levy [33]; Boroujerdi [32]). However, this result applies equally to the case of sets of $N$ irregularly spaced inputs of equal magnitude $A$ per averaging period, conditional on equal values of the product $N \times A$, as illustrated in Figure S4 using various values of $N$, $A$, and $k_{in}$ conditional on $k_{out}$ = 2/hour. Figure S4 was plotted using *Mathematica*® 11.0 software [31] to solve numerically for the function $X(t)$ defined as

$$\mathrm{d}X(t)/\mathrm{d}t \;=\; R_{\mathrm{in}}(A, k_{in}, N) - \; k_{\mathrm{out}}\, X(t), \;\text{ where}$$

$$R_{\mathrm{in}}(A, k_{in}, N) = \; \sum_{j=0}^{n} \sum_{i=0}^{N-1} \Delta(t - i - j\; dt)\;\; A\, k_{\mathrm{in}}\; e^{-k_{\mathrm{in}}(t-i-j\; dt)}$$

in which $A$, $N$, $k_{in}$, and $k_{out}$ were defined above, $T$ = 8 h, $(s)$ denotes a unit-step function increasing from 0 to 1 at time $s$, and the number $n$ of repeated $N$-input cycles is sufficiently large (e.g., 15) to ensure attainment of approximate dynamic equilibrium after $n$ cycles within a selected tolerance (e.g., $10^{-6}$).

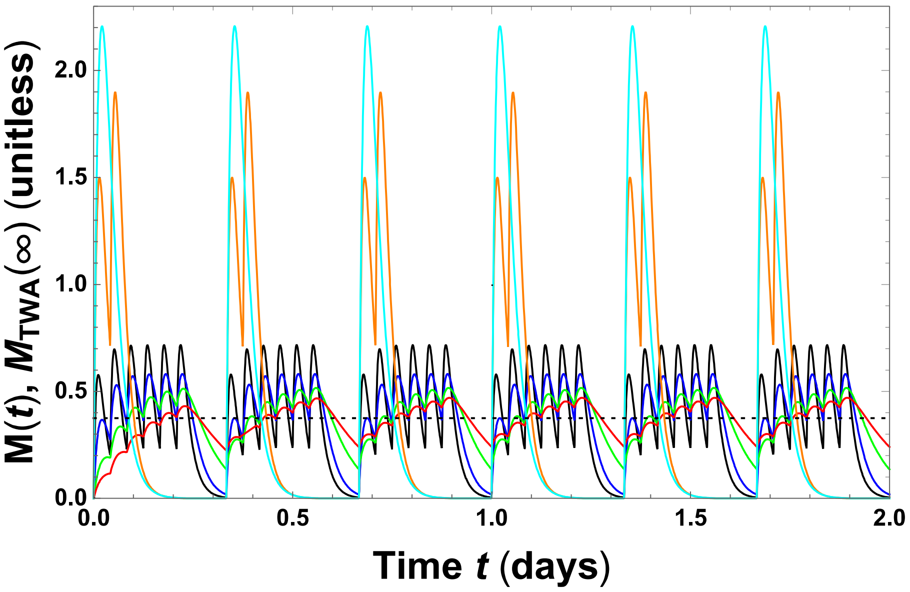


**Figure S4.** Plots of the function $X(t)$ using parameter sets $\{A, k_{in}, N\} = \{1, 3, 6\}$ (black, 3), {1, 1, 6} (blue, 3), {1, 1/3, 6} (green, 4), {1, 1/6, 6} (red, 7), {3, 2, 2} (orange, 2), {6, 1, 1} (cyan, 2), where after each parameter set the list (color, $m$) specifies a corresponding plot color and number $n$ of cycles required to attain dynamic equilibrium within $10^{-6}$. Note that in each case $A \times N = 6$, $k_{out}$ = 2/hour, and $T = 8$ h. The dashed line shows that the TWA value $\bar{X}$ of each plotted function $X(t)$ over averaging period $T$ at dynamic equilibrium has the same value, $\bar{X} = (A \times N)/(k_{out}\ T) = 6/16 =$ 0.375, which is in each case the input-to-output ratio over time $T$.
